# Supplementary material for: Oncolytic BHV-1 Is Sufficient to Induce Immunogenic Cell Death and Synergizes with Low-Dose Chemotherapy to Dampen Immunosuppressive T Regulatory Cells
Source: Cancers (Basel). 2023 Feb 17;15(4):1295. doi: 10.3390/cancers15041295 (PMC9953776; doi:10.3390/cancers15041295)
Supplement: Supplementary file 1 [file cancers-15-01295-s001.zip › cancers-2199386-supplementary.pdf]

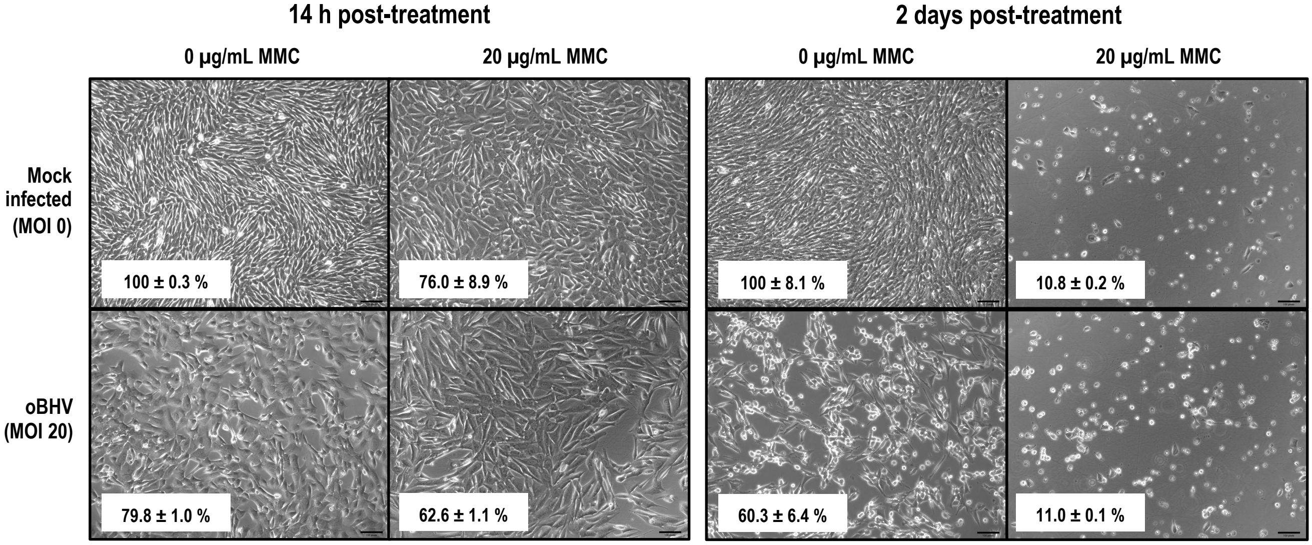

Figure S1: Evaluating optimal conditions for ICD gold standard assay. B16-C10 cells were mock or infected with oBHV at multiplicity of infection (MOIs, plaque forming unit [pfu] per cell) 20. After 1h at 37°C, cells were washed with PBS and media was added back. After 2h incubation at 37°C, media was replaced with fresh media with or without 20 µg/ml of MMC. After 14 h or 2 days, cells were visualized under microscope with magnification 10x, cytopathic effect was evaluated and cellular viability was measured using AlamarBlue assay (Thermo Fisher Scientific), following the manufacturer's instructions. Absorbance was measured using the SpectraMax i3 Multi-Mode Microplate Reader (Molecular Devices). % cellular viability was calculated relative to untreated mock infected cells and is shown as mean ± SEM. Data are shown as representative results from 3 independent experiments with n = 3 per group.

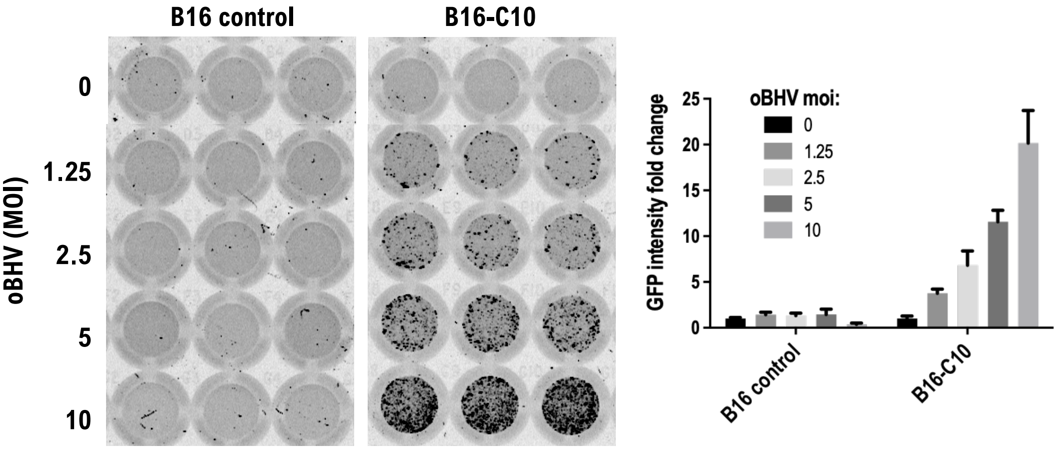

Figure S2. Expression of human nectin-1 (hNectin-1) increases the susceptibility of B16 cells to oBHV. B16 and B16-C10 cells seeded in 96-well plates were infected with 50 µL oBHV at different MOIs for 1 h at 37 °C. Viral inoculum was removed, 100 µL media per well was added back and plates were incubated at 37 °C. Two days post-infection (dpi), plates were scanned on a Typhoon BioAnalyzer (GE Healthcare) to visualize GFP fluorescence as a marker of initiation of virus replication. GFP intensity was quantified and GFP intensity fold change was calculated relative to mock infected cells (MOI 0). Data are shown as representative results from 3 independent experiments with n = 3 per group.

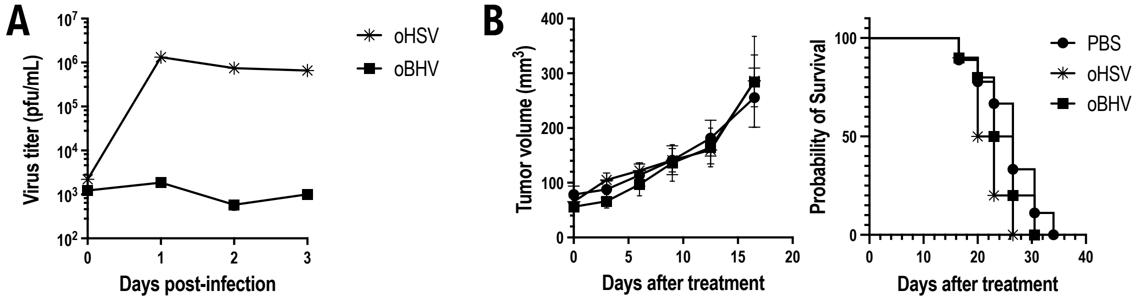

Figure S3. Low-productive infection of oBHV in B16-C10 cells fails to explain low efficacy as monotherapy. (A) B16-C10 cells seeded in 6-well plates were infected with 0.5 mL oBHV or oHSV per well at MOI 5 for 1 h at 37 °C. Cells were washed with PBS and, then, 2 mL media was added back and cells were incubated at 37 °C. Total virus was harvested at 0, 1, 2 or 3 days post-infection (dpi). Samples were freeze/thawed three times and centrifugated at 3,000 rpm for 10 minutes at 4 °C. Supernatant was collected and titrated by serial dilution in serum-free DMEM. For oBHV samples, dilutions were applied to CRIB cells for 1 h at 37 °C and after washing with PBS, DMEM containing 1% FBS and 0.5% methylcellulose was added. For oHSV samples, dilutions were made in serum-free DMEM in the presence of 3 mmol/l hexamethylene bisacetamide (Sigma) and applied to U2OS cells (human osteosarcoma cells, ATCC) for 1 h at 37 °C, and after washing with PBS, DMEM supplemented with 2% human serum (Gibco), 3 mmol/l hexamethylene bisacetamide and 1% FBS was added. At 2-3 dpi, cells were scanned on a Typhoon BioAnalyzer (GE Healthcare), pfu were counted and virus titer calculated. (B) C57BL/6 mice were implanted with B16-C10 subcutaneous tumors (5 x 10^6 cells/mouse). When tumors were palpable (50-100 mm^3), PBS, 2 x 10^7 pfu oHSV or 2 x 10^7 pfu oBHV were administrated i.t. daily for 3 consecutive days. Tumor volumes were measured every 3-4 days until end point (525 mm^3). Average tumor growth and Kaplan-Meier survival curves are shown.

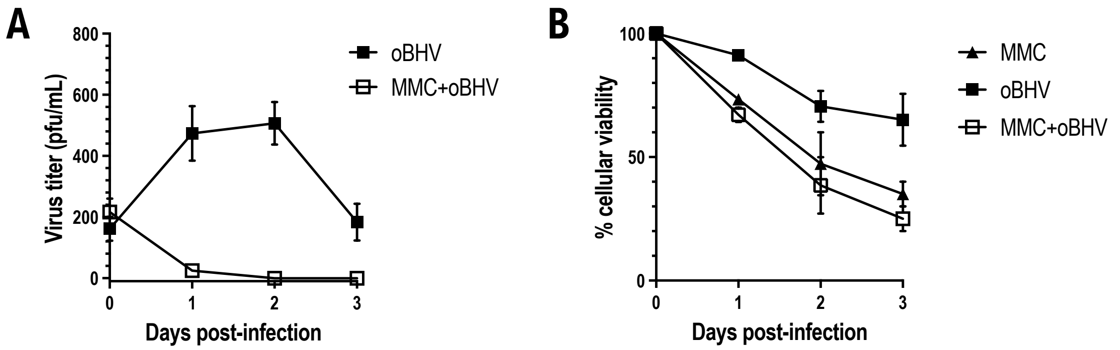

Figure S4. MMC significantly dampens the production of new oBHV particles without altering B16-C10 viability. (A) B16-C10 cells seeded in 6-well plates were infected with 0.5 mL oBHV per well at MOI 10 for 1 h at 37 °C. Cells were washed with PBS, 40 mM citrate buffer (to remove virus particles attached to the cell membrane) and PBS and, then, media was added back. After 2 h incubation, cells were treated with or without MMC (5 µg/ml). Total virus was harvested at 0, 1, 2 or 3 dpi. Samples were freeze/thawed three times and centrifugated at 3,000 rpm for 10 minutes at 4 °C. Supernatant was collected and titrated by serial dilution in serum-free DMEM. Dilutions were applied to CRIB cells for 1 h at 37 °C and after washing with PBS, DMEM containing 1% FBS and 0.5% methylcellulose was added. At 3 dpi, cells were scanned on a Typhoon BioAnalyzer (GE Healthcare), pfu were counted and virus titer calculated. (B) B16-C10 cells seeded were infected with oBHV at MOI 10 for 1 h at 37 °C. Cells were washed with PBS and, then, media was added back. After 2 h incubation, mock and infected cells were treated with or without MMC (5 µg/ml). Cellular viability was quantified at 0, 1, 2 and 3 days post-treatment using AlamarBlue assay and percentage of cellular viability was calculated relative to untreated mock infected cells.

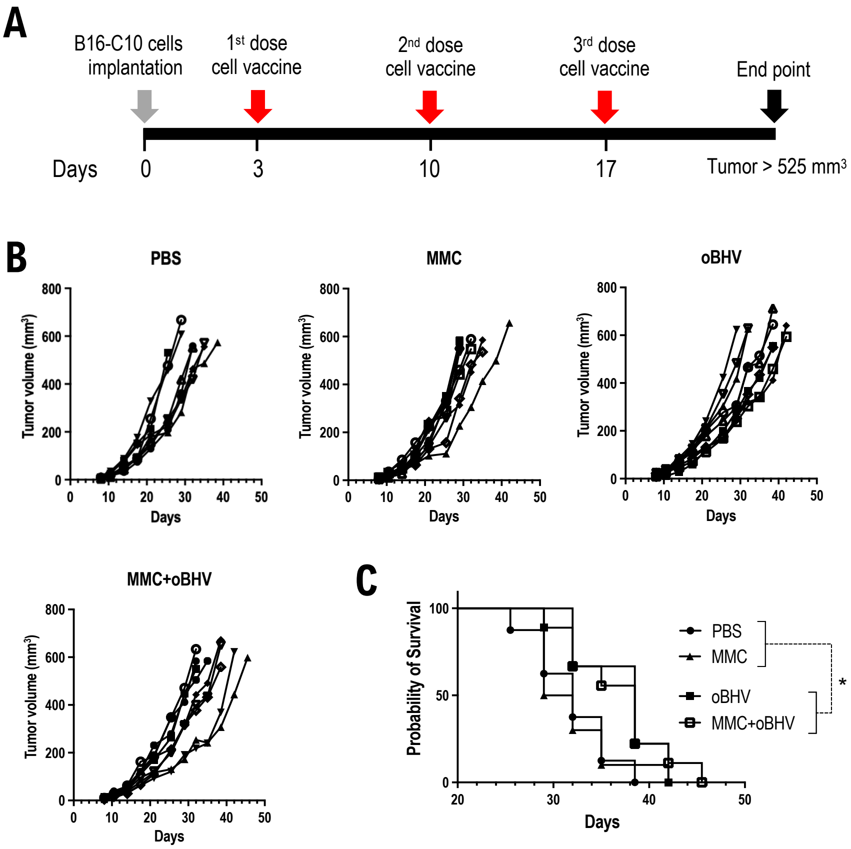

Figure S5: Efficacy of oBHV alone or in combination with MMC as an infected cell vaccine. (A) C57BL/6 mice were implanted with B16-C10 subcutaneous tumors (5×10^6 cells/mouse), and PBS or B16-C10 cell vaccines (3×10^6 dying cells) were administrated subcutaneously on days 3, 10 and 17 post-implantation. To prepare the cell vaccines, B16-C10 cells were treated with MMC, oBHV or MMC+oBHV following the same protocol used for ICD assay (see Figure 2A). Tumor volumes were measured from day 8 every 3-4 days until end point. Tumor volume progression of each mouse in each group (B) and Kaplan-Meier survival curve (C) are shown. All data are pooled from one independent experiment. * $p<0.05$.

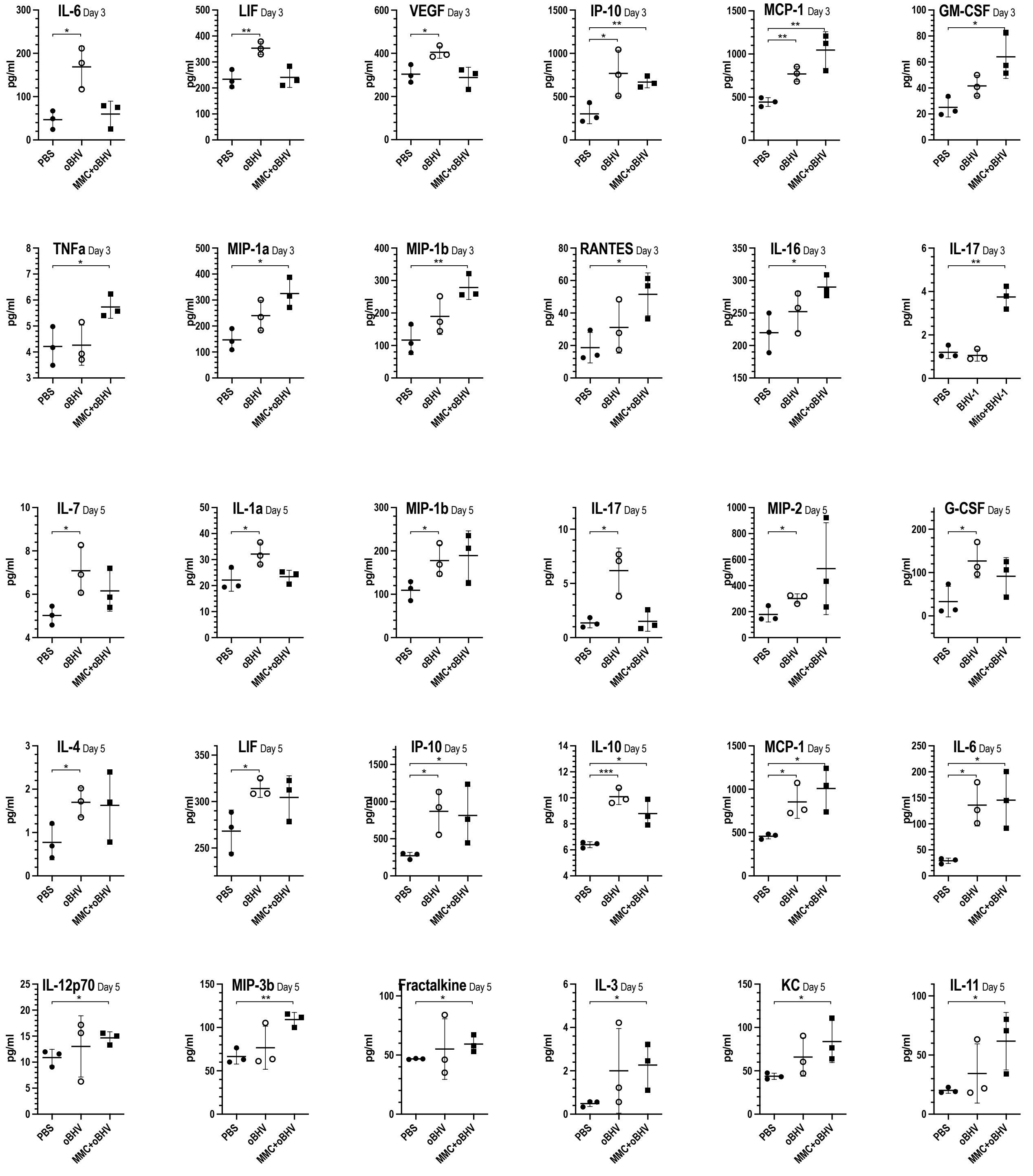

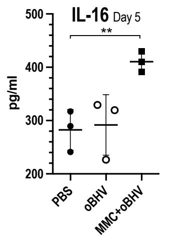

Figure S6. Effect of oBHV treatment with or without MMC on cytokine production in B16-C10 tumors. B16-C10 tumors were treated with PBS, oBHV or MMC+oBHV as described in Figure 6A. On days 3 and 5 tumors were harvested and processing for cytokine quantification via Mouse Cytokine 44-Plex Discovery Assay. * $p < 0.05$, ** $p < 0.01$,*** $p <0.001$. MIP-1a = CCL3, MIP-1b = CCL4, RANTES = CCL5, IP-10 = CXCL10, MCP-1 = CCL2, Fractalkine = CX3CL1, MIP-2 = CXCL-2, MIP-3b = CCL19, KC = CXCL1.
